# Computational Cardiology: The Door to the Future of Interventional Cardiology

**DOI:** 10.1016/j.jacadv.2023.100625

**Published:** 2023-09-16

**Authors:** Yashendra Sethi, Inderbir Padda, Sneha Annie Sebastian, Arsalan Moinuddin, Gurpreet Johal

**Affiliations:** a PearResearch, Dehradun, India; b Government Doon Medical College, HNB Uttarakhand Medical Education University, Dehradun, Uttarakhand, India; c Department of Medicine, Richmond University Medical Center, Staten Island, New York, USA; d Department of Medicine, Azeezia Medical College, Kollam, Kerala, India; e Vascular Health Researcher, School of Sport and Exercise, University of Gloucestershire, Gloucester, United Kingdom; f Valley Medical Center, University of Washington, Seattle, Washington, USA

**Keywords:** artificial intelligence, computational cardiology, fractional flow reserve derived from computed tomography, interventional cardiology, SYNTAX score

**Figure:**
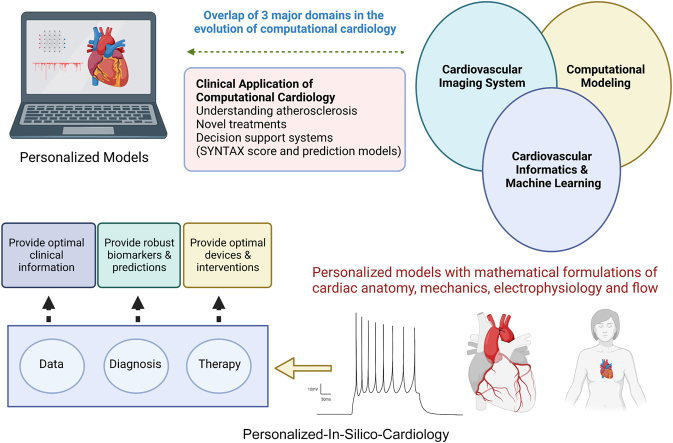

Precision medicine in cardiology has opened up new avenues for phenotypically personalized, integrative, and patient-centered treatment.^1^ Interventional cardiology is not untouched either (Figure 1). With the developments in artificial intelligence (AI), the computational models have revolutionized not only the ‘precision’ of the therapy but also the outcomes. Interventional cardiology, as a specialty, relies on the structural anatomy of the heart, more precisely banking on existing patient data. Computational cardiology allows an extension to the design by predicting outcomes of an intervention in individual patients utilizing integrated patient data sets and models based on physiology and physics (as opposed to population statistics). Further, it satisfies the need for patient-specific models in direct pharmaceutical therapy, device deployment, and surgical interventions.^2^^,^^3^

**Figure 1:**
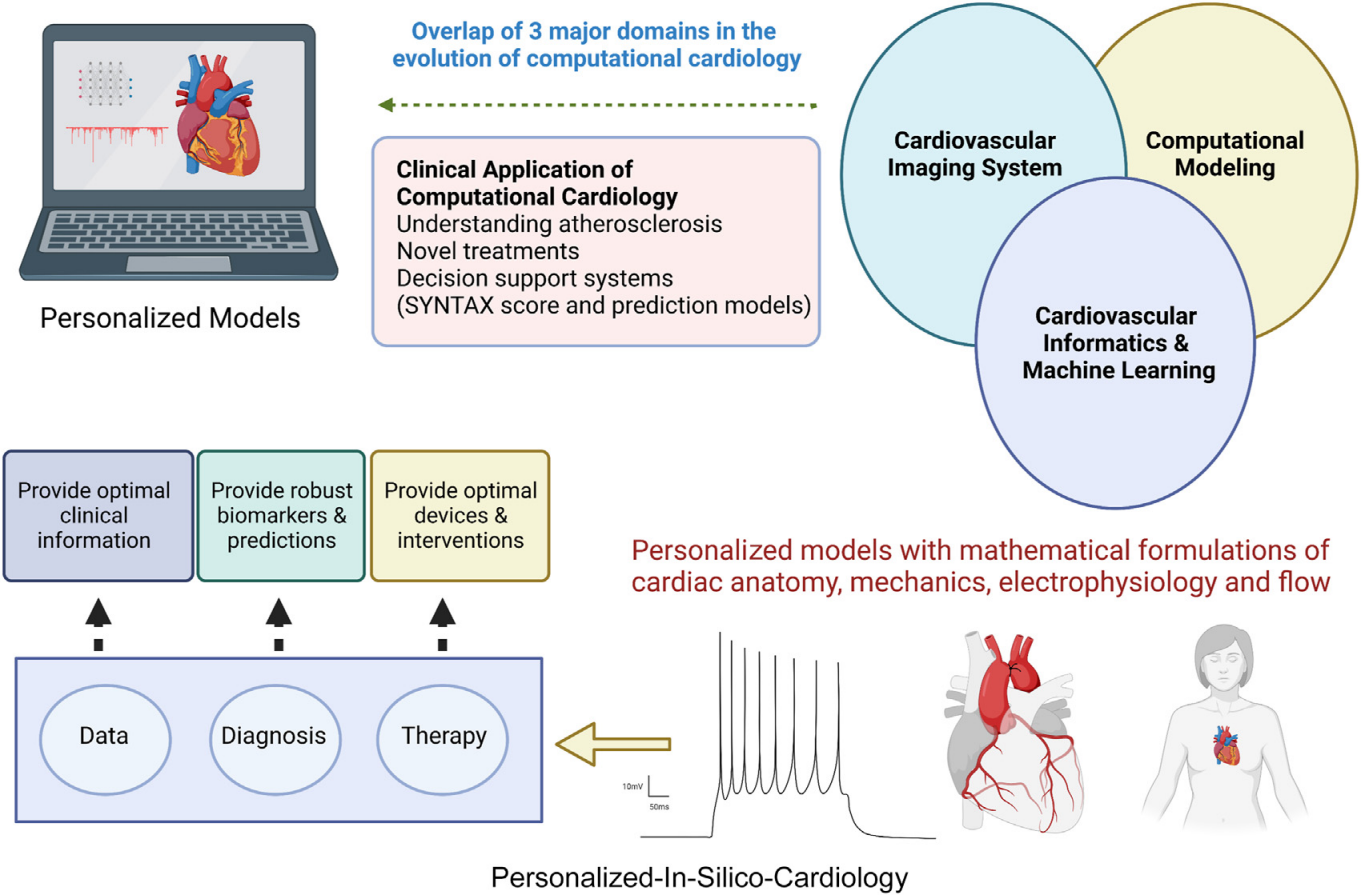
Computationally Empowered Interventional Cardiology The advent of precision medicine and advancements in artificial intelligence have allowed developing personalized models to hone the planning for therapy and predict outcomes, helping to improve patient care in interventional cardiology. The illustration was created using Biorender.

Over the past decade, computational cardiology and AI-based models have been tested for various interventional procedures, including arrhythmias, atrial appendage closure, and postischemic procedures. As such, HeartFlow fractional flow reserve derived from computed tomography (FFRCT) [HeartFlow] and FEops HEARTguide (FEops)—derived from in silico trials—are the 2 applications of computational modeling in contemporary interventional cardiology. 1) HeartFlow FFRCT constructs a 3D model of patients' coronary arteries from static coronary computed tomography images simulating pressure, velocity, and blood flow to estimate the fractional flow reserve. This enables 3-dimensional visualization of defects in the coronary circulation in comparison with the current data and predicts both the outcome and risk/benefit analysis of the intervention. Additionally, HeartFlow FFRCT can be employed to envisage the outcome of coronary stenting, including the residual coronary flow after percutaneous coronary intervention (PCI) or patients with vulnerable plaques, for whom PCI may be of preventative benefit.^2^^,^^3^ 2) The FEops HEARTguide combines digital twin-based predictive stimulation with AI-based anatomical measurements to provide insights into specific patient anatomy like calcium displacement, residual periprosthetic leak, and the occurrence of conduction abnormalities preoperatively in patients undergoing transcatheter aortic valve implantation for severe aortic stenosis.^4^ Trayanova’s lab employed a postinterventional procedure to predict scar distribution on magnetic resonance imaging in post-infarction patients with ventricular tachycardia using accurate conduction velocity maps. They found that during substrate mapping, the integration of noninvasive cardiovascular maps with electroanatomic mapping has the potential to enhance procedural planning and outcomes.^5^ Further, they have developed models for assisting electrophysiological interventions for various arrhythmias.^6^ Of importance, the PREDICT-LAA trial recently suggested that AI-enabled computed tomography-based computational modeling can enhance procedural efficiency for transcatheter left atrial appendage closure as well as its outcomes.^7^

The results are indeed motivating but not without pragmatic obstacles in their implementation. The developing models need to control and ensure the granularity and type of data to be employed, with a plethora of irrelevant variables that have the potential to contaminate the model. Secondly, we need human trials with high internality before their clinical implementation.^8^ Lastly, efforts should be directed to reduce the door-to-balloon time and to find a way to apply these models beyond elective procedures. There have been attempts to use AI-based machine learning algorithms to predict the SYNTAX score (GESS trial) and apply optical coherence tomography/intravascular ultrasound-based computational models to delineate the plaque progression, albeit the limitations with the armory—particularly big data sets, resolution, and penetration of imaging modalities.^8^, ^9^, ^10^

The AI-based computational research currently aims to provide the following 2 strata of admissible evidence: 1) precise imaging of intravascular structures to foresee plaque composition and location, predicting accurate stent placement and expansion; and 2) limiting the development of symptomatic complications from myocardial injury and enhancing the quality of life.^3^^,^^10^ We will need more measures to assemble patient data and enhance our record systems so that every patient has a computational model of their heart ready, even before they face an episode requiring the intervention.

Ultimately, the goal of AI-based computational research should be to inform clinicians about the accuracy of pathologies within the coronary vessels and cardiac structure and limit the procedural time from the onset of pathology to treatment. The future is not far away when a cardiologist planning to do a PCI could use an individualized patient model to underpin the best approach to execute the intervention and the type and size of stent/instrumentation needed.

To conclude, interventional cardiology has seen the development of predictive models for objectively measuring atherosclerosis, predicting the need and type of intervention, calculating fractional flow reserve, and seeking insights into specific patient anatomy including calcium displacement, residual periprosthetic leak, and the occurrence of conduction abnormalities preoperatively. There continue to exist economic and administrative challenges in bringing all these to clinical practice in a scalable manner. Likely, we will need interspecialty collaborative efforts to test and verify the evidence, administrative/government support to seek a national database allowing a pool of individual data, and a vision to shape the future of precision interventional cardiology.

## Funding support and author disclosures

The authors have reported that they have no relationships relevant to the contents of this paper to disclose.

## References

[1] Sethi Y., Patel N., Kaka N. (2023). Precision medicine and the future of cardiovascular Diseases: a clinically Oriented Comprehensive Review. J Clin Med.

[2] Niederer S.A., Lumens J., Trayanova N.A. (2019). Computational models in cardiology. Nat Rev Cardiol.

[3] Hokken T.W., Ribeiro J.M., De Jaegere P.P., Van Mieghem N.M. (2020). Precision medicine in interventional cardiology. Interv Cardiol.

[4] Dugas C.M., Schussler J.M. (2016). Advanced technology in interventional cardiology: a roadmap for the future of precision coronary interventions. Trends Cardiovasc Med.

[5] Aronis K.N., Ali R.L., Prakosa A. (2020). Accurate conduction velocity maps and their association with scar distribution on magnetic resonance imaging in patients with postinfarction ventricular tachycardias. Circ Arrhythm Electrophysiol.

[6] Trayanova N.A., Pashakhanloo F., Wu K.C., Halperin H.R. (2017). Imaging-based simulations for predicting sudden death and guiding ventricular tachycardia ablation. Circ Arrhythm Electrophysiol.

[7] De Backer O., Iriart X., Kefer J. (2023). Impact of computational modeling on transcatheter left atrial appendage closure efficiency and outcomes. J Am Coll Cardiol Intv.

[8] Gray R.A., Pathmanathan P. (2018). Patient-specific cardiovascular computational modeling: diversity of personalization and challenges. J Cardiovasc Transl Res.

[9] Lv R., Wang L., Maehara A. (2023). Combining IVUS + OCT data, biomechanical models and machine learning method for accurate coronary plaque morphology quantification and cap thickness and stress/strain index predictions. J Funct Biomater.

[10] Mittas N., Chatzopoulou F., Kyritsis K.A. (2022). A risk-stratification machine learning framework for the prediction of coronary artery disease severity: insights from the GESS trial. Front Cardiovasc Med.

